# High level of interleukin-33 in cancer cells and cancer-associated fibroblasts correlates with good prognosis and suppressed migration in cholangiocarcinoma

**DOI:** 10.7150/jca.48327

**Published:** 2020-09-23

**Authors:** Supaporn Yangngam, Suyanee Thongchot, Ananya Pongpaibul, Kulthida Vaeteewoottacharn, Somchai Pinlaor, Peti Thuwajit, Seiji Okada, Marcela A. Hermoso, Chanitra Thuwajit

**Affiliations:** 1 Graduate Program in Immunology, Department of Immunology, Faculty of Medicine Siriraj Hospital, Mahidol University, Bangkok 10700, Thailand.; 2 Department of Immunology, Faculty of Medicine Siriraj Hospital, Mahidol University, Bangkok 10700, Thailand.; 3 Siriraj Center of Research Excellence for Cancer Immunotherapy (SiCORE-CIT), Faculty of Medicine Siriraj Hospital, Mahidol University 10700, Thailand.; 4 Department of Pathology, Faculty of Medicine Siriraj Hospital, Mahidol University, Bangkok 10700, Thailand.; 5 Department of Biochemistry, Faculty of Medicine, Khon Kaen University, Khon Kaen 40002, Thailand.; 6 Department of Parasitology, Faculty of Medicine, Khon Kaen University, Khon Kaen 40002, Thailand.; 7 Division of Hematopoiesis, Joint Research Center for Human Retrovirus Infection, Kumamoto University, Kumamoto 860-0811, Japan.; 8 Programa Disciplinario de Inmunología, Instituto de Ciencias Biomédicas, Facultad de Medicina, Universidad de Chile, Santiago, CL 8380453 Chile.

**Keywords:** Cholangiocarcinoma, IL-33, Prognosis, Survival time, Cancer-associated fibroblasts, Migration

## Abstract

Interleukin 33 (IL-33) promotes cholangiocarcinoma (CCA) genesis in a mouse model, however, its function in human CCA has not been clearly understood. This study was aimed to investigate IL-33 level in CCA tissues and its clinicopathological correlations. The results revealed that IL-33 was found in both cancer cells and stromal cancer-associated fibroblast (CAFs) staining patterns which were divided into high (CH) and low level (CL) in cancer cells; and presence (FP) and absence (FA) in CAFs. Kaplan-Meier analysis showed that patients in the CL group were significantly correlated with a short 2-year survival time (*P* = 0.027). The CL/FP group had a shorter survival time compared to the other groups with statistical significance for 2-year (*P* = 0.030) and 5-year (*P* = 0.023) survivals. In contrast, CH/FP patients had significantly greater 2-year (*P* = 0.003) and 5-year (*P* = 0.003) survivals. Univariate and multivariate analysis confirmed that CL/FP was a significantly independent risk factor whereas CH/FP was a significant protective factor in CCA patients. High IL-33 expressing CCA cells had low migration, but they showed increased migration when IL-33 expression was knocked down. The low level of recombinant human IL-33 (rhIL-33) (0.002 - 2 ng/ml) could promote CCA cell migration, in contrast to the suppressive effect at a high dose (20 - 200 ng/ml). In conclusion, the combination of high IL-33 level in cancer cells and CAFs is a potentially good prognosis marker in CCA patients. The *in vitro* migration suppressive effect of IL-33 may be the potential mechanism supporting its role as a good prognostic marker in CCA patients. The obtained results strengthen IL-33 as a promising predictor and therapeutic target for CCA.

## Introduction

Cholagiocarcinoma (CCA) is the second most frequent primary cancer of the hepatic system and the particular incidence of intrahepatic CCA has increased globally over the past few decades 1. The highest incidence of CCA with the prevalence of 85 cases per 100,000 has been found in the northeast part of Thailand where the *Opisthorchis vervirini* liver fluke is endemic 2. The survival rate of CCA patients is poor as most of the patients are diagnosed at a late stage after metastasis occurs 3. Good biomarkers related to cancer progression which can be utilized in the effective management of CCA patinets are still needed 4.

Interleukin 33 (IL-33), an alarmin protein, is produced and released from the epithelial cells when injuries occur 5. The normal function of IL-33 is to enhance wound healing of the epithelium by promoting cell proliferation, increasing reepithelialization and extracellular matrix deposition. IL-33 acts as the proinflammatory cytokine by triggering immune cells expressing ST2 receptors (ST2L) including T helper 2 cells, regulatory T cells and macrophages 6. In cancer, IL-33 is a possible tumor promotion substance by its direct effect on cancer cells to promote cell proliferation, growth, and metastasis, or an indirect effect for remodeling the tumor microenvironment to induce angiogenesis 7. In non-small cell lung cancer (NSCLC), both recombinant IL-33 treated cells and IL-33 overexpressing cells exhibited increased cell growth and invasion both in *in vitro* and *in vivo* experiments by implanting these cells into nude mice 8. Moreover, the blockage of the IL-33 pathway with the ST2 neutralizing antibody or shRNA against ST2, limited the growth and invasion of NSCLC cells 8. In breast cancer (BCA), IL-33 directly promoted ST2-positive BCA cell proliferation and colony formation via induction of the MEK-ERK, JNK-cJun and STAT3 signaling pathways 9. It was observed that cancer proliferation and neovascularization were increased in the 4T1 mouse BCA cell line implanted with IL-33 in mice with accumulation of immune suppressor cells such as myeloid-derived suppressor cells (MDSCs) and Treg with IL-10 and TGF-β immune suppression cytokines 10. Additionally, in colorectal cancer and gastric cancer, IL-33 treatment stimulated several matrix metalloproteases (MMP) including MMP2, MMP3, MMP9 which could promote cancer migration and invasion via the ST2-ERK pathway 11, 12. In contrast, IL-33 has been described as having the ability to eradicate cancer cells by direct induction of cancer apoptosis in a pancreatic cancer cell line 13. Moreover, in IL-33 transgenic mice injected with melanoma and lung carcinoma cell lines, results revealed significantly increased tumor infiltration cytotoxic CD8^+^ T lymphocytes and natural killer cells compared to non-transgenic mice leading to cytolysis-mediated cancer cell death 14. This evidence supports the roles of IL-33 in both promoting and suppressing cancer.

IL-33 has been shown as a poor prognosis marker in several cancers including breast cancer 15, and head and neck squamous cell carcinoma 16. In the opposite way, a high level of serum IL-33 was correlated with a longer survival time in pancreatic and lung cancer patients 17, 18. In a salivary gland tumor, low expression of IL-33 was correlated with perineural invasion and distant metastasis and a shorter survival time in patients 19. In this group of studies, IL-33 is claimed to be a predictive marker for good prognosis.

In CCA, IL-33 has been reported as a cancer promoter in an experimental mouse model 20-22, but its impact in human CCA is still controversial. It is of great interest to explore the effect of IL-33 on human CCA cells and the potential of IL-33 as a prognosis marker and therapeutic target in human CCA. In this study, IL-33 level was investigated in cancer cells and stromal cancer-associated fibroblasts (CAFs) of clinical human intrahepatic CCA samples. The clinicopathological correlations and survival analyses of the patients with their IL-33 levels were performed and the data revealed the combined IL-33 from cancer cells and CAFs as a good prognosis marker in CCA patients. The *in vitro* effect of recombinant IL-33 to suppress CCA cell migration was investigated to partly support the findings in clinical CCA samples that high IL-33 was correlated with good prognosis and long patient survival time.

## Materials and Methods

### Ethical statement and sample subjects

This study was approved by Khon Kaen University Ethics Committee (EC no. HE581456) based on the declaration of Helsinki and the ICH good clinical practice guidelines. All study subjects provided written informed consents prior to the enrollment into the project in which ninety-one CCA samples with their clinicopathological records were obtained from patients who underwent surgery at Srinagarind Hospital, Faculty of Medicine, Khon Kaen University, Thailand.

### Immunohistochemistry

Three-µm slices of paraffin-embedded tissues were deparaffinized at 60^o^C overnight, then dipped in xylene for 5 min followed by dehydration in absolute ethanol and then 95% ethanol. Next, the slides were placed in 0.5 mM EDTA pH 8.0 at 95^o^C in a water bath for 1h. After letting the slides cool down to room temperature, the slides were blocked for endogenous peroxidase in 3% H_2_O_2_ in methanol for 30 min. The slides were blocked in 2% bovine serum albumin (BSA) with 5% rabbit serum in 1X TBS at room temperature for 30 min., then exposed to 1:400 goat anti-human IL-33 antibody (AF3625, R&D System, MN, USA) in 1% BSA. 1X TBS was added and the mixture incubated at 4^o^C overnight in a moist chamber followed by washing with 1X TBST 3 times. Then 1:300 biotin-conjugated rabbit anti-goat secondary antibody (BA-5000, Vector Laboratories, Burlingame, USA) was applied and incubated at room temperature for 1 h. After washings with 1X TBST, ABC reagents (VECTASTAIN^®^ ABC HRP Kit, Vector Laboratories) were applied to the slides and incubated for 30 min at RT in a moist chamber. The signal was developed after the slides were washed 3 times with 1X TBST by DAB (DAKO Agilent, Santa Clara, USA). Subsequently, all sections were counterstained with hematoxylin and mounted. The level of IL-33 in cancer cells was graded by the percentage of positive areas (P) graded as 0 = negative staining, 1 = 1 - 25%, 2 = 26 - 50%, 3 = 51 - 75%, and 4 = 76 - 100%, multiplied by the intensity score (I) as 0 = negative staining, 1 = weak, 2 = mild, and 3 = strong. The total score was calculated by P multiplied by I as an immunohistochemical (IHC) score with a range of 0 - 12 total score. The median score (IHC score = 6) of the overall scoring was set as the cut-off score for low or high IL-33 groups. The score of 0 - 5 was classified as the low expression group while the score of 6 - 12 was classified as the high expression group. The staining intensity of IL-33 in CAFs occurred in an all or none pattern. Therefore, the expression of IL-33 was reported as presence or absence in CAFs. The scores of IL-33 in cancer cells and/or its presence or not in CAFs were analyzed for the clinicopathological parameters and patient survival times.

### Culture of CCA cell lines and primary culture fibroblast

CCA cell lines including KKU-055, KKU-100 and KKU-213 were obtained from the Japanese Collection of Research Bioresources Cell Bank (JCRB Cell Bank) where the cells were tested for identity verification with STR analysis (DNA fingerprinting), negative for bacterial, mycoplasma and fungus contamination. They were checked for mycoplasma in the laboratory of this study before use. All experiments were performed within 20 passages of CCA cells. Cell morphology and cell doubling times were checked in all experiments. CCA cells were maintained in complete medium containing Dulbecco's Modified Eagle's medium high glucose (DMEM) (Gibco, Thermo Scientific, Waltham, USA) containing 100 U/ml penicillin and 100 µg/ml streptomycin (15140122, Gibco, Thermo Scientific) supplemented with 10% fetal bovine serum (FBS) (Gibco, Thermo Scientific) at 37°C in a 5% CO_2_ incubator. The C096 CCA-associated fibroblasts were isolated from fresh CCA tissue and the gene expression profiles were characterized 23. C096 cells were cultured in the same complete media used for CCA cell lines.

### Wound healing migration assay

Approximately 1 - 1.5 x 10^5^ CCA cells were plated and cultured in 24-well plates with complete medium for 24 h and reached approximately 90% confluence. A reference midline was drawn under the plate. Cells were scraped off along the line using a sterile 200-μl pipette tip and the detached cells were washed off three times with serum-free medium. The remaining cells were then treated with 1% FBS DMEM medium, 100 U/ml penicillin and 100 µg/ml streptomycin, either with or without various concentrations of recombinant human IL-33 (rhIL-33) (200-33, PeproTech, NJ, USA). The scraped area indicated by the reference line was recorded at the beginning (0 h) of treatment and every 6 h until 24 h. The efficiency of migration into the scraped area was taken as a measure of wound healing using Image J software and was calculated by the following formula:

% wound healing = (wound space at 0 h - wound space at every 6 h)/([wound space at 0 h] x 100)

### Stable knockdown of IL-33 in CCA cells

KKU-055 cells were plated at 2.5 x 10^5^ cells per well in 6-well plates and allowed to adhere overnight to reach 70 - 80% confluency. IL-33 shRNA plasmid (sc-75333-SH, Santa Cruz Biotechnology, TX, USA) was transfected into cells using Lipofectamine 3000 Reagent (Invitrogen, CA, USA) following the manufacturer's instructions. Briefly, 3.75 µl of Lipofectamine 3000 Reagent was diluted in Opti-MEM Medium before being mixed equally with 0.5 µg of IL-33 shRNA plasmid. After incubating at RT for 15 min, the DNA-lipid complex was added to KKU-055 cells. After 24 h of transfection, the culture medium was replaced with fresh complete medium and cultured for 48 h. The stable clone was selected by sub-culturing transfected cells and culture in complete medium containing 1.5 µg/ml puromycin (sc-205821A, Santa Cruz Biotechnology). After 2 rounds of selection by puromycin, IL-33 KD KKU-055 cells were collected and checked for IL-33 levels using Western blot analysis. Cell morphology was observed under an inverted microscope and cell viability was determined using the CellTiter 96 AQueous Nonradioactive Cell Proliferation Assay Kit (G5421, Promega, WI, USA). The value at 490 nm absorbance was directly proportional to the number of living cells.

### Western blot for IL-33 and ST2 detection in cell lysate and conditioned-medium

Cell lysate was collected from 80 - 90% confluent cells cultured in complete medium. After being trypsinized and washed with cold 1X PBS, cells were lysed in RIPA buffer lysis buffer system (SC-24948, Santa Cruz Biotechnology) on ice for 1h followed by centrifugation at 10,000 rpm at 4^o^C for 10 mins. The supernatant was collected as the cell lysate protein sample. Conditioned-medium (CM) from CCA cell lines and C096 were collected from all cultured cells that had reached 80 - 90% confluence in complete medium and was then replaced with 1% FBS DMEM with 100 U/ml penicillin and 100 µg/ml streptomycin and cultured at 37°C in a 5% CO_2_ incubator for 24 h. After that, the conditioned medium was collected and centrifuged at 3,000 rpm at 4^o^C for 10 mins to get rid of cell debris. The CM was concentrated using Vivaspin® 6, 5 kDa MWCO Polyethersulfone (28-9322-94, GE Healthcare, IL, USA) by centrifugation at 40,000 g for 90 mins at 4^o^C. Protein concentrations of cell lysate and concentrated CM were measured by the Bradford assay using Bio-Rad Protein Assay Dye Reagent (500-0006, Bio-Rad, CA, USA) and prepared to 2 µg/ul in sample buffer containing 10% SDS, 1.0 M Tris-HCl pH 6.8, Glycerol, 0.05%, (w/v) Bromophenol blue and distilled water. After boiling for 5 mins, 30 µg of cell lysate and 250 µg of CM were loaded per lane in 15% and 12% SDS-PAGE for IL-33 and ST2 detection. Proteins were separated at 120 V for 90 mins and transferred to PVDF membrane at 37 mA for 90 mins using the TE 70 Semi-Dry Transfer Unit. Membrane was blocked in 5% skim milk (70166, Sigma-Aldrich, Missouri, USA) in 1 X TBST. Then, the membrane was incubated with 1: 500 goat anti-human IL-33 antibody (AF3625, R&D System) or 1:250 goat anti-human ST2 antibody (AF523, R&D System) at 4^o^C overnight or RT for 2 h. After washing, membrane was incubated with 1:1,000 rabbit anti-goat IgG-HRP (HAF017, R&D System) at room temperature for 1 h. The immunoreactive signals were visualized by Clarity Western ECL (170-5061, Bio-Rad). The β-actin protein level was used as an internal control to determine the equal amounts of loading proteins from cell lysate. The densitometries of IL-33 and ST2L normalized with β-actin were quantified using Image J.

### Statistical analysis

The sample size and statistical power were calculated using the G*Power program (version 3.0.1.9.4). By using Post-hoc test, 91 CCA cases used in this study had power (1-β err prob) = 0.816, effect size = 0.3 (medium effect size), df = 1, α err prob = 0.05. The correlations between clinicopathological parameters and the levels of IL-33 were performed using Chi-square or Fisher's exact test. The Kaplan-Meier Log Rank test was used for survival analysis while univariate and multivariate analyses were analyzed by Cox Regression analysis. Statistics for the* in vitro* studies were compared with controls using the two-tailed Student's t-test. All statistical analyses were performed using SPSS program (version 23.0, IBM). A *P*-value of less than 0.05 was considered statistically significant.

## Results

### Expression of IL-33 in cancer cells and CAFs and clinicopathological correlations

**Figure 1:**
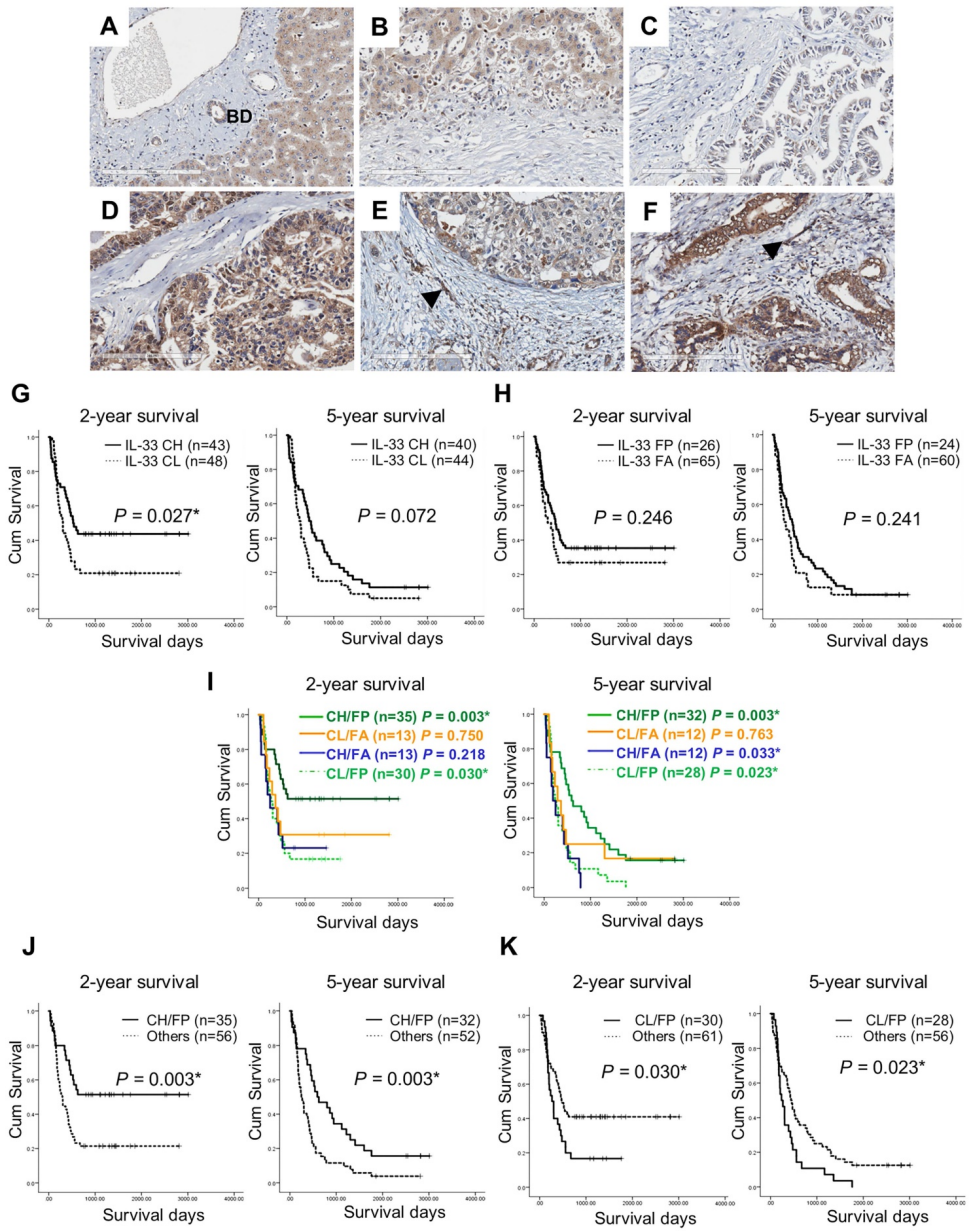
** Immunohistochemistry staining of IL-33 in CCA tissues and Kaplan-Meier analysis (Log Rank test) of IL-33 level and 2-year and 5-year survival in CCA patients.** (**A**) Expression of IL-33 in normal bile duct epithelial cells (BD). (**B**) Absence of IL-33 in the normal hepatic stromal area. (**C-F**) The combined levels of IL-33 in cancer and CAFs groups the samples depending the high (CH) or low (CL) level in cancer cells and either presence (FP) or absence (FA) in CAFs into (**C**) Cancer low/Fibroblast absence: CL/FA, (**D**) Cancer high/Fibroblast absence: CH/FA, (**E**) Cancer low/Fibroblast presence: CL/FP, (**F**) Cancer high/Fibroblast presence: CH/FP. Black arrows represent the presence of IL-33 in CAFs. Original magnification of 200X. Scale bar represents 200 µm. (**G-K**) Kaplan-Meier analysis of different patterns of IL-33 expressions in either cancer cells or CAFs or both using 2-year and 5-year as the cut-off survival times *P-value < 0.05.

The immunohistochemical staining results revealed that cancer cells were the major source of IL-33 in CCA tissues and cytoplasmic localization was detected (Figures 1A - F). The increased expression levels of IL-33 in cancer cells (Figures 1D and 1F) were detected and compared to those in normal bile duct epithelial cells (Figure 1A). The various intensities of IL-33 in cancer cells were detected from low (Figures 1C and 1E) to high (Figures 1D and 1F) while the pattern in stromal fibroblasts was presented as either positive (Figures 1E and 1F, arrows) or negative (Figures 1C and 1D) staining.

Among 91 CCA patients used in this study, 67% (61/91) were male and 33% (30/91) were female (Table 1). The average age of all patients was 57 years old in a range of 38 - 76 years. Sixty-one percent (56/90) of the cases were in stage IV while the remaining 39% (34/90) were in stages I - III. Most of patient tumors were a histological grade of well differentiated 45% (36/80) followed by the papillary type 44% (35/80) and moderately and poorly differentiated 11% (9/80). All 91 patients expressed IL-33 in cancer cells in which 53% (48/91) had high levels and 47% (43/91) had low levels. Interestingly, 71% (65/91) of patients showed positive staining for IL-33 in CAFs whereas it was absent in 29% (26/91) (Table 1). The important clinicopathological parameters including histological grade, staging and periductal invasion had no statistical correlations with IL-33 in cancer cells or CAFs.

The combination of IL-33 levels in cancer cells and CAFs (cancer cells/CAFs) was divided into 4 groups depending high (CH) or low (CL) levels in cancer cells and either presence (FP) or absence (FA) in CAFs included CH/FP, CH/FA, CL/FP, and CL/FA patterns. CCA tissues in which IL-33 staining patterns were CH/FP as the major population of about 39% (35/91) while CH/FA was 14% (13/91) (Table 2). Among CL-CCA cases, 33% (30/91) and 14% (13/91) were CL/FP and CL/FA. Using these combined IL-33 cancer cells/CAFs patterns, however, there were no statistical correlations with any clinical or pathological parameters.

### Combined high IL-33 in cancer cells and the presence in CAFs is a protective marker in CCA patients

Kaplan-Meier analysis revealed that IL-33 levels solely in cancer cells were significantly associated with 2-year survivals (*P* = 0.027) but not statistically significant in 5-year survivals (Figure 1G). Mean survival time of patients with low levels of IL-33 in cancer cells (CL) was 806 ± 158 days compared with 1,747 ± 196 days in patients with a high level of IL-33 in cancer cells (CH). The presence or absence of IL-33 in CAFs had no correlation with survival time (Figure 1H). Using 2-year survival cut-off survival times, CH/FP of IL-33 patterns were significantly correlated with long survival (*P* = 0.003) and CL/FP ratios were related to short survival times (*P* = 0.030) (Figure 1I, 2-year survival). Similar findings for 5-year survival were observed for CH/FP and CL/FP that showed long and short survival times with statistical significance and *P-*values of 0.003 and 0.023 (Figure 1I, 5-year survival). Interestingly, CH/FA also had a significant correlation with short survival time (*P* = 0.033*).* In support, CH/FP showed statistically significant longer 2-year (*P* = 0.003) and 5-year (*P* = 0.003) survival time than other IL-33 expression patterns (Figure 1J). Additionally, the results exhibited that CL/FP had statistically significantly shorter 2-year (*P* = 0.030) and 5-year (*P* = 0.023) survival times than other IL-33 staining patterns (Figure 1K).

Based on univariate analysis by Cox regression as the 2-year survival cut-off, CH/FP was revealed as a significantly independent protective factor with a hazard ratio HR = 0.432; CI = 0.245 - 0.761 (*P* = 0.004), in a similar way that the CL/FP group was revealed as an independent risk factor with HR = 1.755; CI = 1.049 - 2.938 with a statistical significance (*P* = 0.032) (Table 3). A similar phenomenon was observed using the 5-year survival time that indicated that CH/FP was a protective factor with a HR = 0.485; CI = 0.300 - 0.785 (*P* = 0.003) and CL/FP was a risk factor with HR = 1.724; CI = 1.072 - 2.772 (*P* = 0.025).

Moreover, the multivariate Cox proportional hazard regression model indicated that the CH/FP IL-33 pattern was an independently good prognostic factor or a protective factor using a 2-year survival cut-off with HR = 0.392; CI = 0.169 - 0.931 (*P* = 0.034) and HR = 0.392; CI = 0.214 - 0.718 (*P* = 0.002) by the Forced entry and Stepwise methods (Table 4). Tumor staging was an independent risk factor for both analyses with HR = 2.426; CI = 1.461 - 5.159 (*P* = 0.002) and HR = 2.707; CI = 1.464 - 5.005 (*P* = 0.001). In a similar way, using a 5-year survival cut-off, the results indicated that CH/FP was an independent protective factor with HR = 0.384; CI = 0.177 - 0.830 (*P* = 0.015) and HR = 0.460; CI = 0.273 - 0.772 (*P* =0.003) by the Forced entry and Stepwise methods (Table 5) whereas tumor staging was an independent risk factor for both analyses with HR = 1.787; CI = 1.052 - 3.034 (*P* = 0.032) and HR = 1.720; CI = 1.041 - 2.842 (*P*=0.034).

### Low IL-33 enhances CCA cell migration whereas high IL-33 inhibits migration

**Figure 2:**
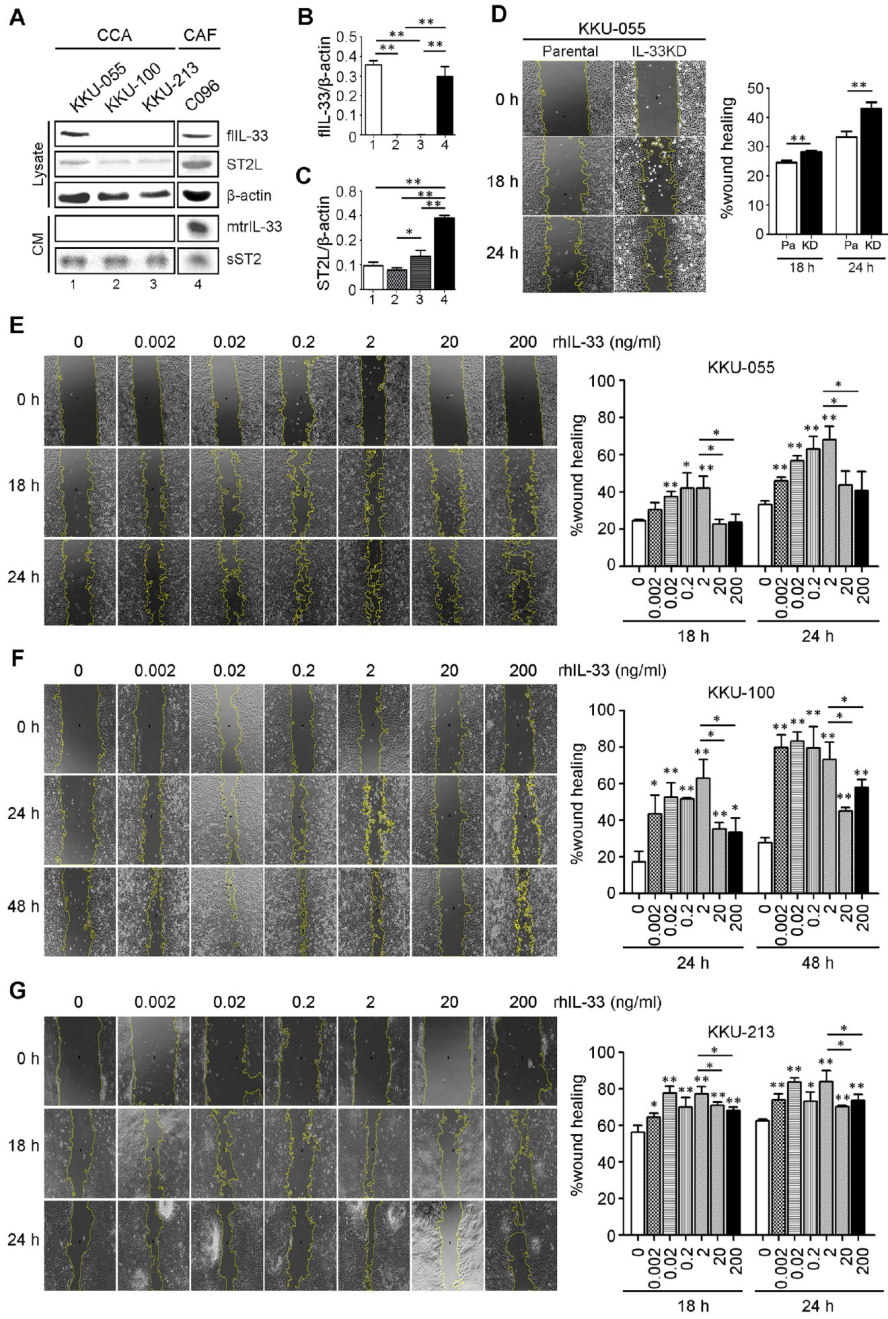
** The effects of rhIL-33 on CCA cell migration.** (**A**) The endogenous or basal expressions of IL-33, ST2L and sST2 in cell lysate and conditioned-medium (CM) of CCA cell lines and CAFs using Western blot analysis. (**B and C**) The densitometry analysis of protein bands in the Western blot analysis. Bar graphs represent mean ± SD of band intensity normalized with the band intensity of β-actin as the internal control. (**D**) Wound healing migration assay of parental KKU-055 compared to IL-33 KD cells at 18 h and 24 h. (**E-G**) The rhIL-33 effect on (**E**) KKU-055 (**F**) KKU-100 and (**G**) KKU-213 migration using the wound healing assay. Graphs represent mean ± SD. *P-value <0.05 and **<0.01 represent the comparisons to those of normal control without rhIL-33 treatments.

The basal levels of IL-33 and receptor ST2 (ST2L) were determined in CCA cell lines (KKU-055, KKU-100 and KKU-213) and a C096 primary culture of CAFs. The results revealed that KKU-055 cells expressed IL-33 intracellularly in full length form (flIL-33), but was not secreted outside the cancer cells in a mature form (mtrIL-33) (Figure 2A). No flIL-33 and mtrIL-33 was detected in KKU-100 and KKU-213 CCA cells. In contrast, C096 CAFs could produce flIL-33 and secrete mtrIL-33 out of the cells. C096 CAF had higher levels of mtrIL-33 than those of KKU-100 and KKU-213 with statistical significance but had the same level to that of KKU-055 cells (Figure 2B). ST2L was expressed in high levels in C096 CAF with statistical significance compared to that in all CCA cell lines which also exhibited the expression of this receptor (Figure 2C). KKU-055 cells had the highest intracellular flIL-33 level among all CCA cell lines that were successfully inhibited by the expression of IL-33 by shRNA against IL-33 with no cytotoxic effects to the cells (Figure S1). IL-33 KD KKU-055 cells had a higher migration ability than parental cells with statistical significance (Figure 2D). Low doses of rhIL-33 (0.002 - 2 ng/ml) enhanced CCA cell migration significantly in a dose dependent manner in KKU-055, KKU-100 and KKU-213 cells but suppressed CCA cell migration at high concentrations (20 - 200 ng/ml) (Figures 2E-G). The intrinsic migration of KKU-055 was significantly lower than KKU-213. Interestingly, the migration of KKU-100 was lower than that of KKU-055, even though not having IL-33 production inside KKU-100.

## Discussion

CCA is an aggressive cancer with a high metastatic rate 2, 3. Finding the interventions to attenuate CCA progression are challenging goals. Having a biomarker to predict the aggressive phenotype is a promising approach to select the best treatment regimen. IL-33 plays an important role in CCA progression in the mouse model 22, however, its role and potential as a prognostic marker in human CCA is unclear. In this work, patients with the combination of high IL-33 in cancer cells and the presence of IL-33 in CAFs had a significantly long survival time. IL-33 in human CCA tissues was proposed as a predictive marker for good survival in CCA patients. The *in vitro* experiments of a high dose IL-33 in cell migration suppression may partly explain high IL-33 in CCA tissues and its correlation with long patient survival time.

The presence of IL-33 could be detected in both cancer cells and CAFs in human CCA tissues. A high level of IL-33 in cancer was observed in this study at approximately 53% in CCA tissues compared to 38% reported in colorectal cancer 24, 41% in hepatocellular carcinoma 25, 59% - 67% in ovarian cancer 26, 27 and 56% in non-small cell lung cancer 28. IL-33 expressing CAFs were reported at around 40% in colorectal cancer 24 and hepatocellular carcinoma 25 which was lower than the 71% in CAFs in CCA tissues found in this work. A high level of IL-33 in breast cancer tissues was correlated with lymph node involvement and metastasis 15; and a high level of IL-33 in tissues revealed short survival in colorectal cancer 24, ovarian cancer 27, glioblastoma 29, renal cell carcinoma 30 and hepatocellular carcinoma 25. This evidence proposes the potential of IL-33 as a poor prognosis marker. In contrast, IL-33 could be a good survival prediction marker as its the high level was correlated with long overall survival time in non-small cell lung cancer 28, malignant salivary gland tumor 19 and large bile duct CCA patients 31.

In this study, the level of IL-33 in CCA tissues was classified into 4 groups based on the level in either cancer cells or stromal CAFs: 1) low IL-33 in cancer cells and absence of IL-33 in CAFs (abbreviated as CL/FA), 2) low IL-33 in cancer cells and presence of IL-33 in CAFs (CL/FP), 3) high IL-33 in cancer cells and absence of IL-33 in CAFs (CH/FA), and 4) high IL-33 in cancer cells and presence of IL-33 in CAFs (CH/FP). The CH/FP showed the longest survival time whereas CL/FP had the shortest survival time compared to the other groups with statistical significance. Univariate and multivariate analysis confirmed that the character of low IL-33 in cancer cells combined with the presence of IL-33 in CAFs was an independent marker for poor prognosis, whereas patients with the presence of IL-33 in CAFs and having a high level of IL-33 in cancer cells had good prognosis. It is possible to conclude that a high level of IL-33 which can be released from either cancer cells or CAFs is correlated with patient good prognosis. This is in concert with the findings in non-small cell lung cancer 28 and malignant salivary gland tumor patients 19 and is the first evidence to exhibit a high IL-33 in CCA microenvironment as a good prognosis marker.

In large bile duct CCA, a high level of intracellular IL-33 was correlated with a less aggressive phenotype 31 which supports the findings in this work in that KKU-055 CCA cells having high IL-33 revealed less cell migration than KKU-213 and KKU-100 CCA cell with low IL-33 intracellularly. Moreover, IL-33 deficient KKU-055 CCA cells could restore the migration ability. These can be explained by the previous report that IL-33 acted as a transcriptional repressor of NF-κB, leading to the decrement of MMPs 32. Moreover, the decrement of NF-κB affected by intracellular IL-33 may inhibit NF-κB-RhoGTPase-mediated cell migration 33. Though the exact mechanism of IL-33 in CCA cell migration is beyond the scope of this study, the above evidence shows the potential mechanisms of intracellular IL-33 in suppressing cell migration.

In the present study, the effect of extracellular IL-33 at a high concentration (20 ng/ml) from either cancer cells or CAFs released into the tumor microenvironment of CCA inhibited cancer cell migration. This is supported by the previous finding that IL-33 ligating to its receptor could inhibit GSK-3β that normally regulates the proliferation and survival of pancreatic cancer cells 34, finally leading to attenuation of cancer progression 35. Moreover, high IL-33 (50 ng/ml) inhibited the growth and promoted apoptosis in colorectal cancer 36 and pancreatic cancer 13. So far, there is no direct evidence of high extracellular IL-33 on cancer cell migration except for the studies in rat cardiac fibroblasts that revealed significant migration suppression by 1-100 ng/ml IL-33 37 and in human trophoblast choriocarcinoma cells for invasion attenuation 38.

Taken all together, high IL-33 in both intracellular and extracellular parts of CCA cells suppressed cell migration. The correlation of high IL-33 in CCA tissues detected in CCA cells or stromal CAFs was correlated with patient long-survival times. Hence, high IL-33 in the tissues is proposed as a biomarker for good prognosis in CCA patients.

## Conclusion

The combined high IL-33 inside the cancer cells with the presence of IL-33 in CAFs is a promising marker for good prognosis in CCA patients. This is partly supported by the *in vitro* findings that high intracellular IL-33 suppressed cancer cell migration and exogenous high IL-33 inhibited cancer cell migration. Though the mechanisms underlying the migration suppressive effect of IL-33 in CCA cell are needed to be further investigated, this study highlights the application of IL-33 level in CCA tissues as a potential predictive biomarker for good prognosis of the patients and strengthens the IL-33 signal pathway as a promising way to treat CCA.

## Supplementary Material

Supplementary figure.

## Figures and Tables

**Table 1:** Clinicopathological correlations and expression of IL-33 in CCA tissues

Parameters	In cancer cells				In CAFs		
	High	Low	*P*-value		Presence	Absence	*P*-value
**Age (n=91)**							
≥ 57 (n=46)	25	21	0.759		33	13	0.947
< 57 (n=45)	23	22			32	13	
**Sex (n=91)**							
Male (n=61)	31	30	0.659		44	17	0.833
Female (n=30)	17	13			21	9	
**Histological grade (n=80)**							
Well differentiated (n=36)	19	17	0.826		24	12	0.463
Moderately and poorly differentiated (n=9)	5	4	1.000		4	5	0.111
Papillary (n=35)	17	18	0.822		29	6	0.050
**Periductal invasion (n=87)**							
Presence (n=8)	6	2	0.272		7	1	0.426
Absence (n=79)	40	39			54	25	
**Staging (n=90)**							
I-III (n=34)	17	17	0.743		25	19	0.695
IV (n=56)	30	26			39	17	
**IL-33 in CAFs (n=91)**							
Presence (n=65)	35	30	0.741				
Absence (n=26)	13	13					

**Table 2:** The 4 patterns of the combined IL-33 in cancer cells and CAFs and clinicopathological correlations

Parameters	In cancer cells and in CAFs				
	CH/FP	CH/FA	CL/FP	CL/FA	*P*-value
**Age (n=91)**					
≥ 57 (n=46)	17	8	16	5	0.674
< 57 (n=45)	18	5	14	8	
**Sex (n=91)**					
Male (n=61)	23	8	21	9	0.949
Female (n=30)	12	5	9	4	
**Histological grade (n=80)**					
Well differentiated (n=36)	13	6	11	6	0.834
Moderately and poor differentiated (n=9)	2	3	2	2	0.227
Papillary (n=35)	14	3	15	3	0.256
**Periductal invasion (n=87)**					
Presence (n=8)	5	1	2	0	0.521
Absence (n=79)	28	12	26	13	
**Staging (n=90)**					
I-III (n=34)	12	5	13	4	0.859
IV (n=56)	22	8	27	9	

The combination of IL-33 levels in cancer cells and CAFs (cancer cells/CAFs) was divided into 4 groups depending the high (CH) or low (CL) level in cancer cells and either presence (FP) or absence (FA) in CAFs including CH/FP, CH/FA, CL/FP, and CL/FA patterns.

**Table 3:** Univariate analysis (Cox regression) analysis of the 4 patterns of the combined IL-33 in cancer cells and CAFs and the associations with 2- and 5-year survival

Parameters	2-year survival				5-year survival		
	HR	95% CI	*P*-value		HR	95% CI	*P*-value
Age	1.059	0.641 - 1.750	0.823		1.064	0.659 - 1.718	0.780
≥ 57 (2-y n=46; 5-y n=42)							
< 57 (2-y n=45; 5-y n=42)							
Sex	0.922	0.536 - 1.585	0.767		0.987	0.593 - 1.644	0.961
Male (2-yrs n=61; 5-yrs n=57)							
Female (2-yrs n=30; 5-yrs n=27)							
Histological grade	0.490	0.281 - 0.855	0.012*		0.429	0.249 - 0.738	0.002*
Non-Papillary (2-yrs n=45; 5-yrs n=44)							
Papillary (2-yrs n=35; 5-yrs n=30)							
Periductal invasion	1.079	0.431 - 2.702	0.872		1.155	0.498 - 2.679	0.738
Presence (2-yrs n=8; 5-yrs n=6)							
Absence (2-yrs n=79; 5-yrs n=74)							
Staging	0.399	0.225 - 0.709	0.002*		0.407	0.236 - 0.703	0.001*
I-III (2-yrs n=35; 5-yrs n=30)							
IV (2-yrs n=55; 5-yrs n=53)							
IL-33 in cancer cells	1.765	1.060 - 2.940	0.029*		1.489	0.918 - 2.416	0.107
High (2-yrs n=48; 5-yrs n=44)							
Low (2-yrs n=43; 5-yrs n=40)							
IL-33 in CAFs	0.726	0.422 - 1.250	0.249		0.703	0.419 - 1.179	0.182
Presence (2-yrs n=65; 5-yrs n=60)							
Absence (2-yrs n=26; 5-yrs n=24)							
IL-33 in cancer/CAFs							
CH/FP	0.432	0.245 - 0.761	0.004*		0.485	0.300 - 0.785	0.003*
Yes (2-yrs n=35; 5-yrs n=32)							
No (2-yrs n=56; 5-yrs n=32)							
CH/FA	1.527	0.775 - 3.001	0.221		1.960	1.042 - 3.683	0.037*
Yes (2-yrs n=13; 5-yrs n=12)							
No (2-yrs n=78; 5-yrs n=72)							
CL/FP	1.755	1.049 - 2.938	0.032*		1.724	1.072 - 2.772	0.025*
Yes (2-yrs n=30; 5-yrs n=28)							
No (2-yrs n=61; 5-yrs n=56)							
CL/FA	1.122	0.552 - 2.278	0.750		0.903	0.464 - 1.758	0.763
Yes (2-yrs n=13; 5-yrs n=12)							
No (2-yrs n=78; 5-yrs n=72)							

**Table 4:** Multivariate analysis (Cox Regression) of the 4 patterns of the combined IL-33 in cancer cells and CAFs and associations with 2-year survival

Parameters	Force entry method				Stepwise method		
	HR	95% CI	*P*-value		HR	95% CI	*P*-value
**Histological grade**	0.55	0.311 - 0.979	0.042		0.573	0.328 - 1.003	0.051
Non-Papillary (n=45)							
Papillary (n=35)							
**Staging**	2.746	1.461 - 5.159	0.002*		2.707	1.464 - 5.005	0.001*
I-III (n=35)							
IV (n=55)							
**IL-33 in cancer cells**	1.210	0.470 - 3.112	0.692				
Presence (n=65)							
Absence (n=26)							
**IL-33 in cancer/in CAFs**							
**CH/FP**	0.392	0.169 - 0.931	0.034*		0.392	0.214 - 0.718	0.002*
Yes (n=35)							
No (n=56)							
**CL/FP**	1.352	0.617 - 2.962	0.451				
Yes (n=30)							
No (n=61)							

**Table 5:** Multivariate analysis (Cox Regression) of the 4 patterns of the combined IL-33 in cancer cells and CAFs and the associations with 5-year survival

Parameters	Forced entry method				Stepwise method		
	HR	95% CI	*P*-value		HR	95% CI	*P*-value
**Histological grade**	0.631	0.373 - 1.067	0.086		0.573	0.328 - 1.003	0.051
Non-Papillary (n=44)							
Papillary (n=30)							
**Staging**	1.787	1.052 - 3.034	0.032*		1.720	1.041 - 2.842	0.034*
I-III (n=30)							
IV (n=53)							
**IL-33 in cancer cells**	1.768	0.712 - 4.389	0.220				
Presence (n=60)							
Absence (n=24)							
**IL-33 in cancer/in CAFs**							
**CH/FP**	0.384	0.177 - 0.830	0.015*		0.460	0.273 - 0.772	0.003*
Yes (n=32)							
No (n=32)							
**CL/FP**	1.735	0.810 - 3.718	0.157				
Yes (n=28)							
No (n=56)							
