# Supplementary material for: High level of interleukin-33 in cancer cells and cancer-associated fibroblasts correlates with good prognosis and suppressed migration in cholangiocarcinoma
Source: J Cancer. 2020 Sep 23;11(22):6571–81. doi: 10.7150/jca.48327 (PMC7545672; doi:10.7150/jca.48327)
Supplement: Supplementary file 1 — Supplementary figure. [file jcav11p6571s1.pdf]

Figure S1 Stable knock down of IL-33 in KKU-055 using shRNA plasmid. (**A**) Cell morphology of parental KKU-055 cells had no change compared with IL-33 knocked down (IL-33KD) cells. Original magnification of 200X and 400X. (**B**) Western blot analysis of full-length IL-33 (flIL-33) level in parental and IL-33KD KKU-055 CCA cells. IL-33 was completely knocked down by IL-33 shRNA. (**C**) The viable cells using the MTS assay of parental and IL-33KD KKU-055 cells. Graph represents mean ± SD of triplicates of one experiment.

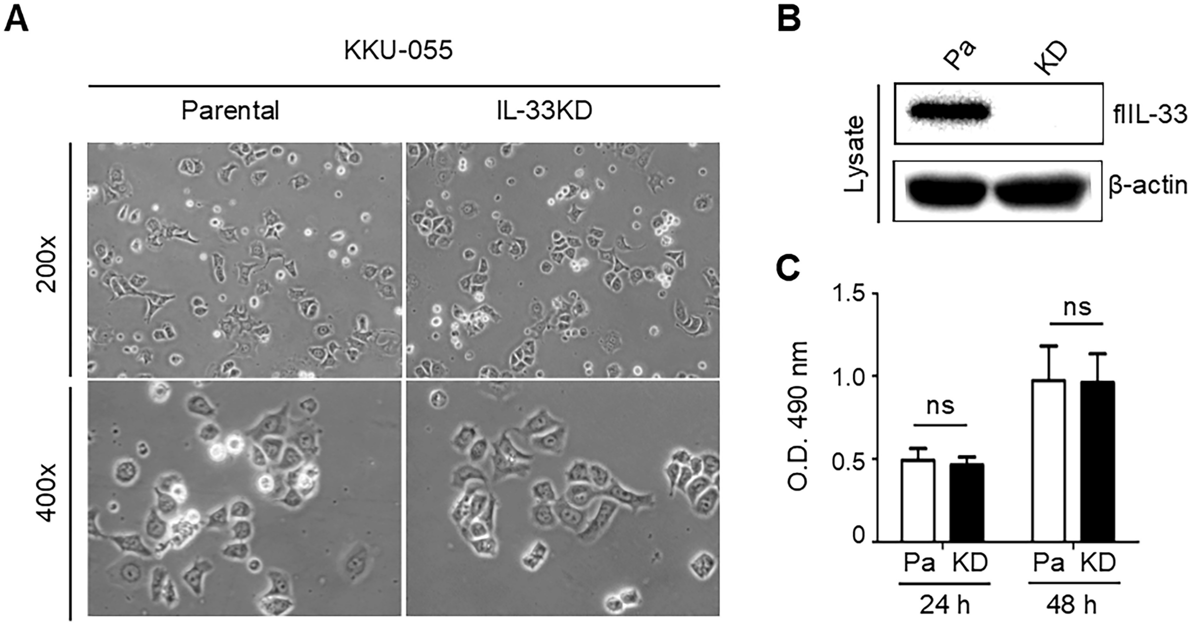

A
KKU-055
Parental
IL-33KD
200x
400x
B
Pa
KD
Lysate
flIL-33
β-actin
C
O.D. 490 nm
1.5
1.0
0.5
0
ns
ns
Pa
KD
Pa
KD
24 h
48 h
